# Epigenetics and Breast Cancers

**DOI:** 10.1155/2012/602720

**Published:** 2012-04-10

**Authors:** An T. Vo, Richard M. Millis

**Affiliations:** Department of Physiology & Biophysics, The Howard University College of Medicine, Washington, DC 20059, USA

## Abstract

Several of the active compounds in foods, poisons, drugs, and industrial chemicals may, by epigenetic mechanisms, increase or decrease the risk of breast cancers. Enzymes that are involved in DNA methylation and histone modifications have been shown to be altered in several types of breast and other cancers resulting in abnormal patterns of methylation and/or acetylation. Hypermethylation at the CpG islands found in estrogen response element (ERE) promoters occurs in conjunction with ligand-bonded alpha subunit estrogen receptor (Er**α**) dimers wherein the ligand ER**α** dimer complex acts as a transcription factor and binds to the ERE promoter. Ligands could be 17-**β**-estradiol (E2), phytoestrogens, heterocyclic amines, and many other identified food additives and heavy metals. The dimer recruits DNA methyltransferases which catalyze the transfer of methyl groups from S-adenosyl-L-methionine (SAM) to 5′-cytosine on CpG islands. Other enzymes are recruited to the region by ligand-ER**α** dimers which activate DNA demethylases to act simultaneously to increase gene expression of protooncogenes and growth-promoting genes. Ligand-ER**α** dimers also recruit histone acetyltransferase to the ERE promoter region. Histone demethylases such as JMJD2B and histone methyltransferases are enzymes which demethylate lysine residues on histones H3 and/or H4. This makes the chromatin accessible for transcription factors and enzymes.

## 1. Introduction

Breast cancers are the most common malignancies and causes of cancer deaths in women worldwide, consisting of approximately 18% of all female cancers. The incidence of breast cancers is much higher in Western than in Eastern countries [1] and geographical variation indicates a significant role of environmental factors in the risks for breast cancers. The Japanese who migrate to Hawaii develop the disease at similar rates as their native counterparts in Hawaii within one or two generations [2]. Upon examining the molecular mechanisms underlying the development and progression of breast cancers, genetic mutations have been an evident cause that has long been established. However, epigenetic mechanisms are now becoming recognized as significant factors in the development of breast cancers. Epigenetic mechanisms coordinate biological processes such as X-chromosome inactivation, position effect variegation, genomic imprinting, RNA interference, and reprogramming of the genome during differentiation and development leading to gene silencing [3]. Defects in any of these functions may cause human disorders including breast cancer. Epigenetic malfunctions are manifested through aberrant methylation and acetylation of genes and histones involved in normal tissue development to activate or silence gene expression. Consequently, abnormal tissue differentiation and growth may result from the loss of crucial cell adhesion proteins and overexcitation of estrogen receptor pathways. Additionally, migration of abnormal cells is increased. Angiogenesis which nourishes tumor growth and important intracellular signal transduction networks such as those for apoptosis, DNA repair, and detoxification are involved. Thus, numerous molecular processes could go awry because of epigenetic malfunctions. Epigenetic mechanisms are strongly influenced by environmental factors such as the chemicals in foods. The occurrences of such epigenetic processes, therefore, suggest that individuals should ingest a balanced diet that includes foods which are known to be protective and supportive while avoiding or limiting exposures to the known risk factors for breast cancers.

## 2. Epigenetic Mechanisms: Methylation and Acetylation

### 2.1. DNA Methylation of CpG Islands and Methylation Imbalance in Cancer Cells

CpG islands are short sequences of genomic DNA with the length of 0.5 kilobase to several kilobases [4] in which the frequency of the linear 5′-CpG-3′ sequence is higher than at other regions of the gene, where “*p*” indicates the phosphodiester bond that connects cytosine and guanine nucleotides [5]. Typical CpG islands are unmethylated and are most commonly found in 5′-regulatory (promoter) regions of many “housekeeping” genes (which are essential for general cell functions) and in some tissue-specific genes [6]. Although a significant portion of CpG islands are in the 5′-untranslated regions and the first exon of genes, they are also found in the 3′-region and within the body of genes. Atypical locations (referring to exonic CpG islands that are not at promoters) are equally if not more prone to methylation [7].

In breast cancers and in many other disease states, hypermethylation of CpG islands results from overactivity of DNA methyltransferases (DNMTs). DNMTs are the main mediators of DNA methylation by catalyzing the transfer of a methyl group from S-adenosyl-L-methionine (SAM) onto the carbon on the 5′-position of CpG dinucleotides. In humans, the primary DNMTs are DNMT1, DNMT3a, and DNMT3b. DNMT1 is the most abundant and functions to maintain the methylation pattern while the other DNMTs serve as the mediators of *de novo* methylation [8]. The consequence of hypermethylation of CpG islands is (reversible) silencing of tumor suppressor genes. Such hypermethylation-induced gene silencing is heritable, that is, inherited by subsequent generations of cells undergoing mitotic divisions. DNA methylation of gene coding regions suppresses gene expression in normal cells [9]. However, in cancer cells, there appears to be widespread hypomethylation of the genomic DNA with hypermethylation localized at the normally unmethylated promoter regions, thereby producing “methylation imbalance” [10]. Methylation is also known to activate the human telomerase reverse transcriptase (hTERT) gene [8], thereby promoting cell immortality in some cancer cells. On the other hand, hypomethylation of CpG islands has been shown to increase expression of some cancer-promoting oncogenes [11].

Genomewide methylation patterns are thought to be reliable indicators of environment-gene interactions. Whereas histone methylation results in short-term inhibition of gene expression, DNA methylation at the CpG islands of promoter regions generates long-term gene silencing and makes the majority of chromatin inaccessible for transcription [12]. The methylation pattern is thought to be determined very early in embryogenesis, at implantation [13]. CpG islands appear to play unique roles in development and differentiation. Although unmethylated CpG islands of gene promoter regions mark sites where genes can be expressed, non-CpG sequences at nonpromoter regions closely associated with transcription start sites may be important for tissue-specific dynamical *de novo* methylation [14]. Methylation of such non-CpG sites has been implicated in somatic cell reprogramming [12] like that which may occur in response to the environment-gene interactions which promote the development of breast and other cancers.

DNA methylation may serve as a marker of breast tumor cell lineage restriction, thereby reflecting the cell type from which a cancer originates and, perhaps, explaining the correlations of histological heterogeneity and prognosis of breast cancers with their DNA methylation profiles [15]. The expression of estrogen receptors in hormone-dependent tumors has also been correlated with the clinical outcomes of cancer patients [16]. The findings of a significant number of hypermethylated genes suggest that the amount of methylation of specific gene clusters may be correlated with the presence of estrogen and progesterone receptors, tumor relapse, and lymph node metastasis [17]. The expression of six genes (RECK, SFRP2, UAP1L1, ACADL, ITR, and UGT3A1) is reported to be significantly correlated with methylation and relapse-free survival; the RECK gene is notable because of its association with the worst cancer prognoses [17]. Moreover, global hypomethylation of breast cancer cell DNA is reported to be associated with a novel methylation pattern. The expression of genes within partially methylated domains in normal breast cells is, paradoxically, inhibited in cancer cells. Hypomethylation of DNA in these regions is found to be associated with repressive chromatin exhibiting an allelic pattern of methylation wherein one allele is DNA-methylated and the other allele contains the histone modifications H3K9me3 or H3K27me3 [18].

### 2.2. Histone Modifications

Histone modifications are catalyzed by several enzymes: Histone acetyltransferases (HATs), histone deacetylases (HDACs), histone methyltransferases (HMTs), and histone demethylases (HDMs). Histone methylation and acetylation to activate or deactivate genes depend on which residue of the histone is modified. For example, trimethylation of lysine 4 on histone H3 (H3K4me3) activates gene transcription but on lysine 9 (H3K9me3) and lysine 27 (H3K27me3) suppress, gene transcription. Methylation of arginine is presumed to also activate transcription. Histone acetylation at lysine residues located at gene promoter regions may activate genes by relaxing and opening the chromatin, thereby providing access to the DNA by transcriptional enzymes and other factors [8].

## 3. Estrogens and Epigenetic Mechanisms in Breast Cancers

### 3.1. The Role of Estrogens and Estrogen Receptors (ERs)

Estrogens have been recognized as the major hormones stimulating the growth and development of breast cancers. The activities of estrogens are mediated by the two main isoforms of intracellular estrogen receptors (ERs): ER*α* and ER*β* which are encoded by the genes ESR1 and ESR2, respectively. These cytoplasm/nuclear ERs have structural characteristics of the nuclear receptor superfamily and they can form homo- or heterodimers when activated by an effective ligand. These dimers can function as transcription factors to regulate gene expression. There are also some ERs located in the plasma and/or organelle membranes associated with G-proteins, tyrosine kinases (e.g., EGFR and IGF-1), and nonreceptor tyrosine kinases. These ERs are involved in signaling cascades to increase second messengers and activate other proteins and kinases [19]. Thus, the activities of ERs involve both genomic pathways to activate or repress transcription, as well as nongenomic pathways via intracellular signaling to control cell cycle progression [20]. Non-nuclear ER-activated mechanisms are reported to have the capacity to induce proliferation of endothelial cells but not of endometrial or breast cancer cells [21].

 ER*α*-mediated transcription appears to increase the risk of breast cancers [22]. Endogenous estrogens synthesized by various body tissues and exogenous estrogens ingested in foods are the most apparent causes of such breast cancers. The primary estrogen in postmenopausal women is estrone (E1) whereas about 70% of the endogenous estrogens is comprised of 17-*β*-estradiol (E2), although E1 and E2 appear to be equally distributed in the sera of premenopausal women [23]. E1 is converted to E2 by catabolism in the liver and 17-*β* hydroxysteroid dehydrogenase activity is reported to be higher in breast tumors than in normal breast tissues [24]. A higher serum level of E2 appears to promote the development of breast cancers in postmenopausal women [24]. However, a clear relationship between serum levels of E1 or E2 and risks for breast cancers in premenopausal women is lacking [25]. These findings suggest the hypothesis that strategies which block hepatic conversion of E1 to E2 may decrease the risk of breast cancers in postmenopausal women.

 The main sources of biosynthetic estrogens include the reproductive organs such as mammary and adipose tissues. Several studies have shown that postmenopausal obese women (above age 55) have an increased risk of breast cancers because of increased estrogen production from aromatization of androgens in peripheral fat tissues [26]. Also, the production of sex hormone-binding globulin among obese women is decreased, hence, possibly raising the level of unbound (free) estrogen to target tissues [26]. Furthermore, Suzuki et al. [26] have shown that the risk of developing estrogen receptor-positive and progesterone receptor-positive (ER+ and PR+) breast cancers is increased in individuals possessing a relatively high body weight. However, this study showed an inverse correlation between body weight and the development of ER+ PR− and ER− PR− tumors. A significant difference between PR+ and PR− tumors in relation to body weight (i.e., PR+ and PR− tumors among obese patients with BMI > 30 kg/m^2^ is reported to be high compared to the proportion among underweight patients with BMI < 18.5 kg/m^2^. These findings suggest the possibility that estrogen, via ER stimulation, gives rise to PR+ tumors [27]. In fact, it has long been established by *in vitro *studies that estrogen regulates the expression of PR via ER in breast cancer cells [28]. These results support the epidemiological findings that postmenopausal status and obesity synergistically increase the risk of breast cancers and that obesity alone confers significant risk for breast cancers. Although some studies have shown that obese postmenopausal women have as much as a 50% greater risk for breast cancers than nonobese women, being overweight/obese is also associated with significantly increased risks of recurrences and deaths from breast cancers regardless of menopausal status [29].

### 3.2. Epigenetic Suppression of ER*α* in Breast Cancers

ER*α* is expressed in about 75% of diagnosed breast tumors (ER*α* positive) and women with such tumors appear to have a better prognosis because of their responsiveness to hormone treatment [30]. On the other hand, women having breast tumors without ER*α* expression (ER*α* negative) are shown to have poorer prognosis and greater malignancy [31]. Several mechanisms have been proposed to explain the cause of suppressing ER*α* expression seen in ER-negative tumors. For example, estrogen withdrawal, hypoxia, overexpression of epidermal growth factor receptor (EGFR) or erythroblastic leukemia viral oncogene homolog 2 (ERBB2), and hyperactivation of mitogen-activated protein kinases (MAPKs) have been suggested [31].

Another mechanism for suppressing the expression of ER*α* is epigenetic silencing via aberrant methylation of the ER*α* promoter. The loss of expression of ER*α* and/or E-cad (cell-cell adhesion molecule) genes appears to result from aberrant methylation of CpG islands [31]. ER*α* and E-cad both play important roles in maintaining the normal differentiated state of the mammary gland epithelium. Neither gene is methylated in normal breast epithelia; however, methylation has been observed in all tumor stages with greater incidences during progression from ductal carcinoma in situ (DCIS) to metastatic tumors [31]. These results suggest that epigenetic silencing of the ER*α* and E-cad genes may occur prior to invasion and may, therefore, increase as cells acquire invasiveness, and metastatic potential. Greater frequency of coincident methylation of CpG islands is a result of the accumulation of epigenetic “hits” that contribute to gene silencing. Consequently, ER*α* loss during breast cancer progression is associated with poor histological differentiation, high growth fraction, and adverse clinical outcomes and may represent a key mechanism facilitating hormone resistance [31]. Similarly, loss of E-cad expression is associated with loss of differentiation, increased invasiveness and high metastatic potential which decreases patient survival. Loss of the ER*α* and E-cad expressions which support normal cell growth could be an evolutionary adaption to stop tumor growth; however, these adaptations may not have advanced to the stage of stopping growth before the cancer cells have established their invasive and metastatic potential.

ER suppression by microRNAs (miRNAs) has also been shown. MiRNAs are small noncoding RNAs that suppress gene expression posttranscriptionally by base pairing to 3′-untranslated regions (3′UTR) of target mRNAs. A study by Di Leva et al. [30] has demonstrated a loop regulation between miR-221-222 and ER*α*. ER*α* appears to bind to miR-221-222 and recruit the corepressors NCoR and SMRT to the miR-221-222 promoter region and, thereby, reduce their expression. Overexpression of miR-221 and -222 in ER*α*-positive breast cancer tissues results in ER*α* suppression at the post-transcriptional level and also suppresses the expression of different tumor suppressors such as CDKN1B, CDKN1C, BIM, PTEN, TIMP3, DNA-damage-inducible transcript 4, and FOXO3. Consequently, miR-221-222 promotes high proliferation and estrogen-independent growth. Interestingly, miRNA expression is regulated by ER*α* and these findings suggest that the activity of this regulatory loop may confer proliferative advantage and migratory activity to breast cancer cells and promote the transition from ER-positive to ER-negative tumors [30].

### 3.3. Estrogens and Histone Demethylation: Modifying Histones via Demethylation by ER*α* and JMJD2B

JMJD2B is a histone lysine-specific demethylase and enrichment of H3K9me3 distal to the ER binding site functions as a repressive mark of transcription. Upon activation of E2, a process that is dependent on JMJD2B, the methylation level of H3K9me3 may be decreased [32]. This mechanism for JMJD2B to decrease the level of H3K9me3 may enhance the transcriptional activation of oncogenes and antiapoptotic genes. The mechanism of E2 and JMJD2B is as follows: E2 induces JMJD2B expression in an ER*α*-dependent manner and available JMJD2B is then recruited to ER*α* target sites, interacts with ER*α* and SWI/SNF, and demethylates H3K9me3 to facilitate transcription of ER responsive genes including MYB, MYC, CCND1. Knockdown of JMJD2B impairs E2-ER*α* cell proliferation and tumor formation, and its deletion from mammary epithelial cells produces delayed mammary gland development in mice [32]. These findings suggest that JMJD2B is necessary for the full extent of ER*α* transcriptional activity.

### 3.4. Estrogens and Histone Acetylation: E2-ER*α* Dimers and HATs Modify Histones by Acetylation

E2 works by binding to the ER*α* monomer and releasing chaperone proteins that change the conformation of the ER. Binding of E2 causes ER*α* monomers to dimerize and enter the nucleus to act as transcription factors [33]. ERs work in conjunction with HATs and the histone lysine-specific demethylase JMJD2B which acts as a coactivator to open the chromatin for transcription. E2-ER*α* dimers recruit ATP-dependent chromatin remodeling complexes (SWI/SNF) and histone modifying enzymes to estrogen-responsive promoters [20]. This gives rise to an increase in breast cell divisions which have the potential to promote tumor growth and increase replicating errors in cancer-related genes. Histone modifying enzymes include the HATs, namely, p160, CBP, p300, and pCAF, which are recruited sequentially. Certain HATs also work to facilitate binding to RNA polymerase II and priming the promoter for multiple rounds of transcription [20].

 In addition to the endogenous estrogen E2, various sources of xenoestrogenic compounds such as birth control pills, estrogen replacement therapy, and phytoestrogens are known to activate the ER*α* monomer. These phytoestrogenic ligands, which are known to promote cancers, seem to work by binding to the ER*α* monomer whereas those that inhibit cancers appear to do so by binding to the ER*β* monomer [34]. ER*β* is reported to have opposite effects to that of ER*α*, but the mechanisms remain unclear [34]. These observations imply that cancer tumor growth promotion and inhibition by phytoestrogens may depend on the relative expressions of ER*α* and/or ER*β*, as well as of the homo- and heterodimers ER*αα*, ER*ββ*, and ER*αβ* in specific tissue types. These dimers may be differentially expressed in normal breast tissue and breast cancer cells [35]. Because phytoestrogens appear to have greater affinity for the ER*β* than the ER*α* monomer, inhibition of tumor growth may be the predominant effect of phytoestrogens in breast cancers. However, the role of ER*β* is not as well studied as that of ER*α* and its mechanism of action needs further investigation.

## 4. Food Ingredients and Epigenetic Mechanisms in Breast Cancers: Folate/Methionine, Betacarotene/Arachidonic Acid, Heterocyclic Amines PhIP and Food Additive BHA

### 4.1. Folate, Methionine and DNA Methylation

Because DNA methylation requires methyl group donors such as SAM, it is necessary to have a source of methyl groups. Two main sources of methyl groups found in foods are methionine and folate. Methionine is an essential amino acid found in poultry, fish, and dairy products while folate is an essential nutrient found in fruits and vegetables [36]. Numerous studies suggest that DNA methylation may be influenced by diet. Diets low in methionine and folate or high in methyl group antagonists such as ethanol result in aberrant methylation patterns of DNA, including global hypomethylation and methylation of normally unmethylated CpG sites [36]. Global genomic hypomethylation can result from decreased levels of SAM whereas hypermethylation of unmethylated CPG sites can be produced by increases in the activities of DMNTs [37]. In a study of 304 African-American women diagnosed with breast cancers during 1995–1998 living in Tennessee, significant positive correlations were reported for low methionine intake with unknown methylation status of ER*α*-positive tumors and high ethanol consumption with unmethylated ER*α*. On the other hand, there was no significant data showing a correlation between methyl-deficient diets and methylation of the ER*α* gene [36]. These findings suggest that methyl-deficient diets may result in hypomethylation of ER*α* genes and, thus, give rise to ER*α*-negative tumors. Another study demonstrated similar results, showing a trend toward decreased methylation with increasing ethanol intake and a trend toward increased methylation with increasing dietary folate [38]. Because ethanol inhibits the absorption of folate in the intestine and interferes with hepatic release of folate, these findings suggest that the primary carcinogenic mechanism of alcohol intake may be interference with epigenetic regulation through disruption of one-carbon metabolism [38]. It, therefore, seems advisable to insure adequate folate and methionine intake and to limit ingestion of alcohol to decrease the risks for breast cancers.

### 4.2. Beta-Carotene, Arachidonic Acid, and DNA Hypomethylation: Promotion of Angiogenesis by Upregulating Tyrosine Kinase VEGFR-2 Receptor (KDR) Expression Via Demethylation of DNA CpG Islands in the KDR Gene

Angiogenesis is essential for tumor growth. Bevacizumab (Avastin) is the first antiangiogenesis drug approved by the US Food and Drug Administration (FDA) in 2008 for metastatic breast cancers but recently found by an FDA panel to be ineffective at increasing the survival of breast cancer patients [39]. Bevacizumab inhibits production and release of VEGF-A, the most potent paracrine stimulator of angiogenesis [29] and activator of the tyrosine kinase VEGFR-2 receptor (KDR), a coactivator of angiogenesis. Beta-carotene (BC) is reported to be proangiogenic [40]. Kiec-Wilk et al. [41] have reported that BC, present in brightly pigmented red, orange, and yellow fruits and vegetables, as well as arachidonic acid (AA), an omega-6 fatty acid present in vegetable oils, may promote angiogenesis by increasing expression of the KDR in endothelial cells. Moreover, it is reported that incubation of endothelial cells with physiological concentrations of BC (3–10 *μ*M) or AA (3 *μ*M) decreases the global DNA methylation of endothelial cells, as well as methylation of the KDR promoter region. These findings suggest that hypomethylation of the KDR promoter region is a likely mechanism for upregulation of KDRs in endothelial cells by BC and AA. KDR, being an important protein in chemotaxis, differentiation, and angiogenesis [41], suggests roles for BC and AA in breast cancers. The omega-3 fatty acids found in animal meats like chicken and egg yolks promote cellular uptake of BC and AA. Thus, avoidance of high-fat diets appears to be a reasonable strategy for inhibiting the angiogenesis associated with breast cancers. Moreover, BC is known to be stored in adipose tissue, thereby providing a plausible explanation for the predilection for breast cancers in obese women. It is noteworthy that, in ovariectomized mice, tumor VEGF levels in blood and adipose tissues have been reported to be higher during a period of high-fat compared to a period of low-fat dietary treatments. This finding suggests that both high body fat content and high fat diets may have the potential to increase VEGF levels, the substrates for KDRs and angiogenesis.

### 4.3. PhIP in Cooked Meat: PhIP Acts Like E2 to Induce Gene Expression via an ER*α* Mechanism

PhIP is a phenylimidazole pyridine and one of three mutagenic and carcinogenic heterocyclic amines (HAs) produced during the cooking of meats such as beef, pork, and chicken. The concentration of PhIP in these meats increases with the degree of heat applied when cooking; for example, burnt meat has the highest concentration of PhIP but even small concentrations of PhIP in cooked meats are thought to have physiological effects. Exposure to PhIP has been shown to increase cell proliferation in mammary gland terminal end buds, suggesting that PhIP promotes tumorigenesis [42]. A relatively low level of PhIP in breast milk has been demonstrated to exert estrogenic and mitogenic effects in humans via a MAPK pathway [43]. PhIP appears to bind, specifically albeit at a lower affinity than E2, to the ER*α*-ligand binding domain and, therefore, competes with E2 at its binding site, thereby suggesting an epigenetic mechanism for PhIP, akin to that of E2, in breast cancers [44]. Interestingly, the PhIP phase I metabolites, N_2_-OH-PhIP, MeIQx, and IFP, are reported to inhibit the activation of ER*α*. Treatment of MCF-7 breast cancer cells with PhIP and these metabolites significantly inhibits ER*α* transcription activity at a much lower level than treating with PhIP alone. Hence, although PhIP is carcinogenic, these PhIP metabolites are anti-estrogenic [42]. This suggests that dietary constituents have both activating and inhibiting effects on hormone-sensitive tissues such as some breast cancers and the risks and development of breast cancers may result either from a defect in the mechanisms for metabolizing PhIP or from overload of PhIP.

### 4.4. Food Additives: Bisphenol (Antioxidant t-Butylhydroxyanisole, BHA) Is Estrogenic

More than 90 types of chemicals and food additives exert estrogenic activity to stimulate cell growth [45]. Bisphenol A, also known as antioxidant t-butylhydroxyanisole (BHA), is the most extensively used antioxidant in the food industry. BHA is used in fats and oils, fat-containing foods, confectionaries, essential oils, and food-coating materials such as metal cans and waxes. BHA is also often found in polycarbonate plastics (recycle symbol 7) like in some sports drink and infant nursing bottles. BHA is an environmental estrogen because it is a synthetic chemical the actions of which are similar to estrogen. More specifically, BHA is considered as an epigenetic carcinogen because it causes cell proliferation via epigenetic events [46]. In a study testing the two food additives BHA and o-phenyl phenol (OPP), the assay of ER competitive *in vitro* binding to human ER*α* and ER*β* showed that BHA had the capacity to compete with E2. On the other hand, the capacity of OPP was too small to calculate. However, both BHA and OPP induced a decrease in gene expression of ER*α* and an increase in that of PRs in a time-dependent manner. These effects were similar to that of E2, although much higher concentrations were required for BHA and OPP than E2 [45]. In the case of BHA, the danger is that it is widely used. Wear and tear, harsh detergent, and heat can cause BHA to leach out of bottles into the fluid contained therein. Studies conducted in Europe and Japan have reported that BHA can migrate from plastic polyvinyl chloride stretch plastic wraps to food. BHA has also been detected in recycled cardboard (source probably from thermal fax paper which contains BHA) and in “virgin” paper products [47, 48]. It is, therefore, important to use stainless steel bottles or plastic without BHA and avoid reusing or heating BHA-containing products. These findings suggest that constant exposure to BHA containing products and excessive food additives could result in accumulation in the body to increase the risk of breast cancers.

## 5. Heavy Metals and Their Epigenetic Mechanisms in Breast Cancers

### 5.1. Aluminum and Cadmium

Aluminum (Al) is a heavy metal element that is widely used in foods, cosmetics, and other products of human consumption. Al is shown to be carcinogenic by a mechanism akin to that of estrogens; hence, Al is known as a “metalloestrogen.” Al binds to ERs and triggers the expression of genes found on estrogen-responsive elements (EREs). There is evidence that certain salts of Al such as those found in antiperspirants can remain in applied areas of the axillae and breasts for prolonged periods if not washed well, thereby providing the potential for continuous exposure to Al and enhancement of the risks for breast cancers [11]. Cadmium (Cd) is a similar metalloestrogen with a mechanism of action that involves inducing the expression of heat shock proteins, Hsp 22 and Hsp 27. Treatment of MCF-7 human breast cancer cells, which express ER*α* with either E2 or with Cd appears to increase the expressions of Hsp 22 and Hsp 27 [11]. These heat shock proteins are known to form homo- or heterodimers, and high-molecular-weight complexes are shown to be present in breast cancer cells; however, the exact structure and function of these dimmers remain unclear [49]. According to the United States Department of Labor, there are numerous occupations and work environments that expose workers to toxic heavy metals [50]. Cd is an extremely toxic metal commonly found in industrial workplaces, particularly where ores are processed or smelted, thereby putting a large group of metal workers at risk for breast cancers, perhaps, by epigenetic mechanisms involving ER*α* responsiveness to heat shock proteins. Several deaths from acute exposure have occurred among welders who have unsuspectingly welded on Cd-containing alloys or with silver solders. 

### 5.2. Arsenic, Selenium, Chromium, and Nickel

Certain heavy metals have not been shown to play a role in breast cancers but have been demonstrated to act on the same regulatory enzymes previously shown to play important roles in breast cancers. Arsenic (As) competes with DNMT for SAM, potentially limiting the availability of SAM to be used by DNMT to catalyze methylation of CpG islands. Such limitation could result in hypomethylation and reactivation of (silenced) tumor suppressor genes. Common sources of exposure to higher-than-average levels of As include occupations near or in hazardous waste sites and areas with high levels naturally occurring in soil, rocks, and water. Thus, individuals engaged in a wide variety of occupations such as miners, construction, and waste workers are at risk for breast cancers. Similarly, for detoxification, selenium (Se) also requires SAM. Se may, therefore, compete with As and DNMT, thereby also contributing to DNA hypomethylation [11]. Chromium (Cr) is another heavy metal found in raw onion, romaine lettuce, beef, chicken, liver, and many other foods. Cr is an essential mineral and has been shown, at some dosages associated with over-the-counter supplementation, to promote health by supporting normal cholesterol, blood glucose, and insulin levels [51]. However, Cr is also reported to be carcinogenic at high concentrations such as those which may occur from over-supplementation. Cr recruits HDAC1 and DNMT1 to promoters such as the CYP1A1 promoter and forms a complex that recruits binding protein 1 and inhibits the expression of CYP1A1 [11]. CYP1A1 is an important detoxifier that metabolizes carcinogens such as polycyclic hydrocarbons (PHs) and polycyclic amines (PAs) [11]. PAs such as PhIP can increase the risk of breast cancers by binding to and activating ER*α*, and the PhIP metabolites N_2_-OH-PhIP, MeIQx, and IFP inhibit the activation of ER*α* [42]. It, therefore, appears that by decreasing the expression of CYP1A1, Cr may limit the metabolism of PAs, cause PAs to accumulate, induce binding of PAs to ER*α*, and either activate or block activation of ER*α*, depending on the PA metabolite profile. Calcium chromate, chromium trioxide, lead chromate, strontium chromate, and zinc chromate are human workplace carcinogens known to increase the incidence of lung cancers among workers in industries that produce chromate and manufacture pigments containing chromate [50]. The heavy metal element Nickel (Ni) seems to induce carcinogenic effects by inducing hypermethylation of H3K9, inhibiting DNMT and inhibiting histone H2A, H2B, H3, and H4 acetylation which silence tumor suppressor genes [11]. Ni appears to bind differentially to various parts of histones. Ni binding to histone H4 is reported to inhibit histone lysine acetylation, thereby inducing DNA hypermethylation [11].

## 6. Anticancer Effects of Foods

Several of the active compounds in foods work epigenetically to decrease the risk of breast cancers [8]. Enzymes involved in DNA methylation and histone modifications have been shown to be altered in several types of cancers resulting in abnormal patterns of methylation and/or acetylation. Many cancer drugs work by altering the activity and expression of these enzymes. Interestingly, some components of natural foods have similar actions on these enzymes and therefore produce anticancer effects.

### 6.1. Tea Polyphenols, EGCG

Epigallocatechin-3-gallate (EGCG) is a polyphenol in teas that humans ingest. EGCG is reported to block DNMT1-induced methylation of the CpG islands present in many genes, including tumor suppressor genes, in a concentration-dependent manner [52]. EGCG seems to directly inhibit, by hydrogen bonding, the key nucleotide cytosine from entering the DMNT1 active site, thereby, effectively preventing DNA methylation. Consequently, EGCG reactivates tumor suppressor genes and inhibits tumor promoter genes such as hTERT, which increases with hypermethylation [8]. Such inhibition of hTERT is significant because of reports of higher levels of hTERT in breast cancers than in other cancers and of a positive correlation of hTERT with telomerase activity, a key enzyme in cell immortalization [53].

### 6.2. Genistein: An Isoflavone Found in Lupin, Kudzu, Psoralea, Fava, and Soybeans

Genistein is an antioxidant and phytoestrogen, known to inhibit DNMT1/3a/3b and to contribute to inactivating histone H3K9me3 and repressing hTERT expression in human breast cancer cells [8]. The estrogenic effects of genistein are thought to be mediated by an ER*β* pathway [34]. Cappelletti et al. [34] have shown that treatment of hormone-sensitive (T47D) and hormone-independent (BT20) cell lines with genistein results in increased expression of ER*β*2 mRNA, modulated by the ER*β*2 isoform, thereby inhibiting estrogen-promoted cell growth. These findings suggest that the potential benefits of genistein as a chemotherapeutic or prevention agent for breast cancers are likely mediated by an epigenetic mechanism related to expression of ER*β*2 mRNA. Concerns have been raised about the overall benefits of soy products because of the estrogenic and antiestrogenic properties of their isoflavones. Genistein's anticancer and procancer effects have been attributed to its biphasic properties [54]. The estrogenic effect has been observed when administration of genistein at a low dose has induced growth of ER-positive breast cancer cells [55]. Genistein binds to ERs and inhibits cytochrome P450 CYP1A1 which, in turn, increases the production and release of E2 [56]. Studies have produced an anti-estrogenic effect of genistein by inhibiting the enzyme 17*β*-hydroxysteroid oxidoreductase type 1 (HSOR-1) that is necessary for E2 secretion from the ovaries in premenopausal women. Genistein may also be essential for the reduction of E1 to E2 in adipose and other tissues [55]. Thus, the pro- and anti-cancer effects of genistein appear to depend on the effects of genistein on the production of estrogens.

In intact mice fed estrogen, genistein appears to produce tumor growth inhibition [54]. Studies have also shown that isoflavone supplements do not affect breast tissue density in premenopausal women but may decrease breast tissue density in postmenopausal women [61]. Genistein is also reported to decrease cell proliferation and to induce apoptosis, prevent DNA mutation, and inhibit angiogenesis in breast cancer cells [8]. These novel findings on the epigenetic mechanisms of genistein may help explain the discrepancy of breast cancer risk in Western and Eastern countries and the changes in the risk among Asians and Asian-Americans. Epidemiological studies have indicated that about one in eight women in the United States is at risk for developing breast cancers while the risk is fivefold less in women in Japan and China [55]. Among Chinese- and Japanese-Americans, the incidence of breast cancer was found to be about 50% lower in Asian-born women and 25% lower in US-born women than in US-born Caucasians [57]. Furthermore, migrant studies have shown that Asian women who immigrated to the USA and adopted a Western lifestyle developed a risk for breast cancers comparable to that of Caucasian women within two generations [58]. These studies suggest that environmental factors may play a more significant role than genetic factors in breast cancer risk difference between Asian and Caucasian women. An important environmental factor is soy product consumption, which is widely ingested in Asian countries, where the incidence of breast cancers is substantially lower than in Western countries [59, 60]. Interestingly, reports have indicated that Asian women living in Asia have up to 40% lower serum estrogen levels than Caucasian women living in the USA or Britain [55]. This could be attributed to the fact that the consumption of phytoestrogens, particularly soy products, is higher in Asia than in Western countries [59–61]. A more specific study performed by Yamamoto et al. [62] looked at various soy products commonly consumed in Japan and breast cancer risk. This population-based cohort study showed that the consumption of miso soup and isoflavones, but not of soyfoods, was inversely associated with the risk of breast cancers. Furthermore, this association did not change substantially after adjustment for potential confounders, including reproductive history, family history, smoking, and other dietary factors. These correlations were observed in premenopausal women but were reported to be even stronger in postmenopausal women [62].

### 6.3. Sulforaphane: An Isothiocyanate Rich in Broccoli, Cabbage, and Kale

Like genistein, sulforaphane (SFN) also inhibits DNMTs in both MCF-7 and MDA-MB-231 breast cancer cells. This inhibition of DNMTs is reported to induce site-specific CpG demethylation of the first exon of the hTERT gene resulting in both dose- and time-dependent repression of gene transcription [8].

## 7. Conclusions

**Figure 1:**
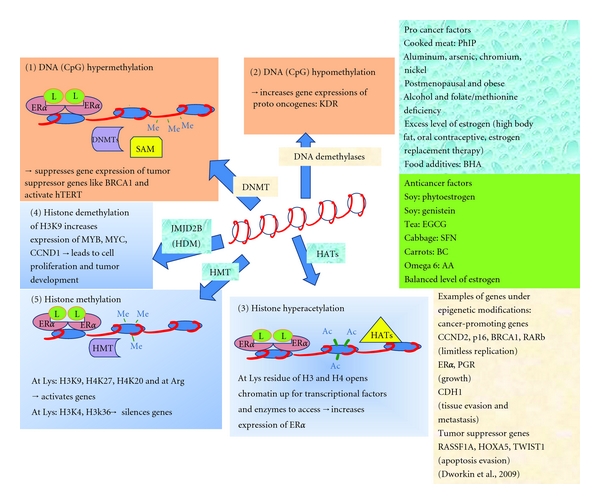
Five mechanisms for epigenetic alterations in breast cancers. Each alteration involves many enzymes but the main players to cause methylation or acetylation are shown by arrows. These are not separate mechanisms and the enzymes do not act alone. Several enzymes act at a promoter simultaneously. (1) Hypermethylation at the CpG islands found in estrogen response element (ERE) promoters. When ligand- (L-) bonded ER*α* dimerizes, the complex (L-ER*α* dimer) acts as transcription factor and binds to the ERE promoter. Ligands could be E2, phytoestrogen, PhIP, and so forth. The dimer may recruit DNMTs which catalyze the transfer of methyl groups from SAM to 5′-cytosine on CpG islands. (2) Other enzymes could be recruited to the region by L-ER*α* dimers which activate DNA demethylases to act simultaneously to increase gene expression of protooncogenes and genes involved in cell growth. (3) L-ER*α* dimer may also recruit SWI/SNF and HATs to the ERE promoter region. HATs include p160, CBP, p300, pCAF. (4) Histone demethylases such as JMJD2B and (5) histone methyltransferases (HMTs) are enzymes which acetylate lysine residues on H3 and or H4 to open up the chromatin for other transcription factors and enzymes. Enzymes/cofactors: DNA methyltransferases (DNMTs), s-adenosylmethionine (SAM), histone acetyltransferases (HATs), histone methylases (HDM) JMJD2B, histone methyltransferases (HMTs), and DNA demethylases (member of MBD, methyl CpG-binding domain, family proteins).

Five mechanisms for epigenetic alterations in breast cancers are summarized in Figure 1. Each alteration involves many enzymes inducing methylation or acetylation which are not separate mechanisms and the enzymes do not act alone. Several enzymes act at a promoter simultaneously such as hypermethylation at the CpG islands found in estrogen response element (ERE) promoters. When ligand- (L-) bonded ER*α* dimerizes, the L-ER*α* dimer complexes act as transcription factors and bind to the ERE promoters. Ligands could be E2, phytoestrogen, PhIP, and so forth. The dimer may recruit DNMTs which catalyze the transfer of methyl groups from SAM to 5′-cytosine on CpG islands. Other enzymes could be recruited to the region by L-ER*α* dimers which activate DNA demethylases to act simultaneously to increase gene expression of proto-oncogenes and genes involved in cell growth. L-ER*α* dimers may also recruit SWI/SNF and HATs to the ERE promoter region. Histone demethylases such as JMJD2B and histone methyltransferases HMTs are enzymes which demethylate histones, for example, lysine residues on histones H3 and/or H4 to make the chromatin accessible to transcription factors and enzymes.

## References

[1] Key TJ, Verkasalo PK, Banks E (2001). Epidemiology of breast cancer. *Lancet Oncology*.

[2] McPherson K, Steel CM, Dixon JM (2000). ABC of breast diseases: breast cancer-epidemiology, risk factors, and genetics. *British Medical Journal*.

[3] Veeck J, Esteller M (2010). Breast cancer epigenetics: from DNA methylation to microRNAs. *Journal of Mammary Gland Biology and Neoplasia*.

[4] Bird AP (1986). CpG-rich islands and the function of DNA methylation. *Nature*.

[5] Millis RM (2011). Epigenetics and hypertension. *Current Hypertension Reports*.

[6] Esteller M (2002). CpG island hypermethylation and tumor suppressor genes: a booming present, a brighter future. *Oncogene*.

[7] Nguyen C, Liang G, Nguyen TT (2001). Susceptibility of nonpromoter CpG islands to de novo methylation in normal and neoplastic cells. *Journal of the National Cancer Institute*.

[8] Meeran SM, Ahmed A, Tollefsbol TO (2010). Epigenetic targets of bioactive dietary components for cancer prevention and therapy. *Clinical Epigenetics*.

[9] Dworkin AM, Huang THM, Toland AE (2009). Epigenetic alterations in the breast: implications for breast cancer detection, prognosis and treatment. *Seminars in Cancer Biology*.

[10] Baylin SB, Herman JG, Graff JR, Vertino PM, Issa JP (1997). Alterations in DNA methylation: a fundamental aspect of neoplasia. *Advances in Cancer Research*.

[11] Mishra S, Dwivedi SP, Singh RB (2010). A review on epigenetic effect of heavy metal carcinogens on human health. *The Open Nutraceuticals Journal*.

[12] Cedar H, Bergman Y (2009). *Epigenetic Silencing during Early Lineage Commitment*.

[13] Michaelson-Cohen R, Keshet I, Straussman R, Hecht M, Cedar H, Beller U (2011). Genome-wide de novo methylation in epithelial ovarian cancer. *International Journal of Gynecological Cancer*.

[14] Straussman R, Nejman D, Roberts D (2009). Developmental programming of CpG island methylation profiles in the human genome. *Nature Structural and Molecular Biology*.

[15] Dedeurwaerder S, Fumagalli D, Fuks F (2011). Unravelling the epigenomic dimension of breast cancers. *Current Opinion in Oncology*.

[16] Thomas C, Gustafsson JA (2011). The different roles of ER subtypes in cancer biology and therapy. *Nature Reviews Cancer*.

[17] Hill VK, Ricketts C, Bieche I (2011). Genome-wide DNA methylation profiling of CpG islands in breast cancer identifies novel genes associated with tumorigenicity. *Cancer Research*.

[18] Hon GC, Hawkins RD, Caballero OL (2012). Global DNA hypomethylation coupled to repressive chromatin domain formation and gene silencing in breast cancer. *Genome Research*.

[19] Hatae J, Takami N, Lin H, Honda A, Inoue R (2009). 17beta-Estradiol-induced enhancement of estrogen receptor biosynthesis via MAPK pathway in mouse skeletal muscle myoblasts. *The Journal of Physiological Sciences*.

[20] Moggs JG, Orphanides G (2001). Estrogen receptors: orchestrators of pleiotropic cellular responses. *EMBO Reports*.

[21] Wu Q, Chambliss KL, Oltmann S (2010). Non-nuclear estrogen receptor *α* signaling promotes cardiovascular protection but not uterine or breast cancer growth in mice. *Journal of Clinical Investigation*.

[22] Ali S, Coombes RC (2000). Estrogen receptor *α* in human breast cancer: occurrence and significance. *Journal of Mammary Gland Biology and Neoplasia*.

[23] Slemenda C, Longcope C, Peacock M, Hui S, Johnston CC (1996). Sex steroids, bone mass, and bone loss: a prospective study of pre-, peri-, and postmenopausal women. *Journal of Clinical Investigation*.

[24] Clemons M, Goss P (2001). Estrogen and the risk of breast cancer. *The New England Journal of Medicine*.

[25] Kaaks R, Berrino F, Key T (2005). Serum sex steroids in premenopausal women and breast cancer risk within the European Prospective Investigation into Cancer and Nutrition (EPIC). *Journal of the National Cancer Institute*.

[26] Suzuki R, Rylander-Rudqvist T, Ye W, Saji S, Wolk A (2006). Body weight and postmenopausal breast cancer risk defined by estrogen and progesterone receptor status among Swedish women: a prospective cohort study. *International Journal of Cancer*.

[27] Parrella P (2010). Epigenetic signatures in breast cancer: clinical perspective. *Breast Care*.

[28] Horwitz KB, McGuire WL (1978). Estrogen control of progesterone receptor in human breast cancer. Correlation with nuclear processing of estrogen receptor. *Journal of Biological Chemistry*.

[29] Gu J-W, Young E, Patterson SG (2011). Postmenopausal obesity promotes tumor angiogenesis and breast cancer progression in mice. *Cancer Biology and Therapy*.

[30] di Leva G, Gasparini P, Piovan C (2010). MicroRNA cluster 221-222 and estrogen receptor *α* interactions in breast cancer. *Journal of the National Cancer Institute*.

[31] Nass SJ, Herman JG, Gabrielson E (2000). Aberrant methylation of the estrogen receptor and E-cadherin 5′ CpG islands increases with malignant progression in human breast cancer. *Cancer Research*.

[32] Kawazu M, Saso K, Tong KI (2011). Histone demethylase JMJD2B functions as a co-factor of estrogen receptor in breast cancer proliferation and mammary gland development. *PLoS ONE*.

[33] Kumar V, Chambon P (1988). The estrogen receptor binds tightly to its responsive element as a ligand-induced homodimer. *Cell*.

[34] Cappelletti V, Miodini P, Di Fronzo G, Daidone MG (2006). Modulation of estrogen receptor-beta isoforms by phytoestrogens in breast cancer cells. *International Journal of Oncology*.

[35] Hayashi S-I, Eguchi H, Tanimoto K (2003). The expression and function of estrogen receptor *α* and *β* in human breast cancer and its clinical application. *Endocrine-Related Cancer*.

[36] Zhu K, Davidson NE, Hunter S (2003). Methyl-group dietary intake and risk of breast cancer among African-American women: a case-control study by methylation status of the estrogen receptor alpha genes. *Cancer Causes & Control*.

[37] Lopatina NG, Vanyushin BF, Cronin GM, Poirier LA (1998). Elevated expression and altered pattern of activity of DNA methyltransferase in liver tumors of rats fed methyl-deficient diets. *Carcinogenesis*.

[38] Christensen BC, Kelsey KT, Zheng S (2010). Breast cancer DNA methylation profiles are associated with tumor size and alcohol and folate intake. *PLoS Genetics*.

[39] United States Food and Drug Administration FDA begins process to remove breast cancer indication from Avastin label. http://www.fda.gov/newsevents/newsroom/pressannouncements/ucm237172.htm.

[40] Dembinska-Kiec A, Polus A, Kiec-Wilk B (2005). Proangiogenic activity of *β*-carotene is coupled with the activation of endothelial cell chemotaxis. *Biochimica et Biophysica Acta*.

[41] Kiec-Wilk B, Razny U, Mathers JC, Dembinska-Kiec A (2009). DNA methylation, induced by *β*-carotene and arachidonic acid, plays a regulatory role in the pro-angiogenic VEGF-receptor (KDR) gene expression in endothelial cells. *Journal of Physiology and Pharmacology*.

[42] Bennion BJ, Cosman M, Lightstone FC (2005). PhIP carcinogenicity in breast cancer: computational and experimental evidence for competitive interactions with human estrogen receptor. *Chemical Research in Toxicology*.

[43] Creton SK, Zhu H, Gooderham NJ (2007). The cooked meat carcinogen 2-amino-1-methyl-6-phenylimidazo[4,5-b]pyridine activates the extracellular signal regulated kinase mitogen-activated protein kinase pathway. *Cancer Research*.

[44] Lauber SN, Ali S, Gooderham NJ (2004). The cooked food derived carcinogen 2-amino-1-methyl-6-phenylimidazo[4,5-b] pyridine is a potent oestrogen: a mechanistic basis for its tissue-specific carcinogenicity. *Carcinogenesis*.

[45] Okubo T, Kano I (2003). Studies on estrogenic activities of food additives with human breast cancer MCF-7 cells and mechanism of estrogenicity by BHA and OPP. *Yakugaku Zasshi*.

[46] Williams GM (1986). Epigenetic promoting effects of butylated hydroxyanisole. *Food and Chemical Toxicology*.

[47] Ozaki A, Yamaguchi Y, Fujita T, Kuroda K, Endo G (2004). Chemical analysis and genotoxicological safety assessment of paper and paperboard used for food packaging. *Food and Chemical Toxicology*.

[48] Lopez-Cervantes J, Paseiro-Losada P (2003). Determination of bisphenol A in, and its migration from, PVC stretch film used for food packaging. *Food Additives and Contaminants*.

[49] Sun X, Fontaine JM, Bartl I, Behnam B, Welsh MJ, Benndorf R (2007). Induction of Hsp22 (HspB8) by estrogen and the metalloestrogen cadmium in estrogen receptor-positive breast cancer cells. *Cell Stress and Chaperones*.

[50] United States Department of Labor (2011). Heavy metals. http://www.osha.gov/SLTC/metalsheavy/index.html.

[51] National Institute of Health http://ods.od.nih.gov/factsheets/chromium.

[52] Li Y, Tollefsbol TO (2010). Impact on DNA methylation in cancer prevention and therapy by bioactive dietary components. *Current Medicinal Chemistry*.

[53] Kirkpatrick KL, Clark G, Ghilchick M, Newbold RF, Mokbel K (2003). hTERT mRNA expression correlates with telomerase activity in human breast cancer. *European Journal of Surgical Oncology*.

[54] Messina MJ, Loprinzi CL (2001). Soy for breast cancer survivors: a critical review of the literature. *Journal of Nutrition*.

[55] Bouker KB, Hilakivi-Clarke L (2000). Genistein: does it prevent or promote breast cancer?. *Environmental Health Perspectives*.

[56] Shertzer HG, Puga A, Chang C (1999). Inhibition of CYP1A1 enzyme activity in mouse hepatoma cell culture by soybean isoflavones. *Chemical and Biological Interactions*.

[57] Stanford JL, Herrinton LJ, Schwartz SM, Weiss NS (1995). Breast cancer incidence in Asian migrants to the United States and their descendants. *Epidemiology*.

[58] Thomas DB, Karagas MR (1987). Cancer in first and second generation Americans. *Cancer Research*.

[59] Parkin DM, Whelan SL, Ferlay J, Raymond L, Young J (1997). *Cancer Incidence in Five Continents*.

[60] Hirose K, Imaeda N, Tokudome Y (2005). Soybean products and reduction of breast cancer risk: a case-control study in Japan. *British Journal of Cancer*.

[61] Messina MJ, Persky V, Setchell KD, Barnes S (1994). Soy intake and cancer risk: a review of the in vitro and *in vivo* data. *Nutrition and Cancer*.

[62] Yamamoto S, Sobue T, Kobayashi M (2003). Soy, isoflavones, and breast cancer risk in Japan. *Journal of the National Cancer Institute*.

